# Ultrasound-guided Placement of a Foley Catheter Using a Hydrophilic Guide Wire

**DOI:** 10.5811/cpcem.2017.12.37045

**Published:** 2018-03-14

**Authors:** Ryan Joseph, Mark Huber, Ben Leeson, Kimberly Leeson

**Affiliations:** Texas A&M University, Department of Emergency Medicine, Corpus Christi, Texas

## Abstract

Acute urinary retention is a common problem in the emergency department. Patients can present in significant distress, necessitating the placement of a urinary catheter. Foley catheter placement can be difficult to accomplish depending on the etiology of the retention and the degree of the obstruction. In the case presented here, we used ultrasound guidance, a guidewire, and a Foley catheter to successfully relieve a patient’s urinary retention after multiple failed attempts.

## INTRODUCTION

There are many causes of acute urinary retention including medications, neurologic disorders, and bladder outlet obstruction. We will focus here on mechanical, or anatomic, bladder outlet obstruction, which in and of itself has many different etiologies, including, meatal stenosis, urethral stricture, bladder neck stricture, and benign prostatic hypertrophy.^1^ Typically, obstructive symptoms include hesitancy, a sensation of incomplete bladder emptying, diminished urinary stream, and post-void urinary dribbling.^2^ The treatment of choice for bladder outlet obstruction is urethral catheterization, and this has traditionally been accomplished using a 16 or 18 French Foley catheter.^3^ If this fails, the next step would be to use a 16 French coudé tip catheter, or Tiemann catheter. This is a semi-rigid catheter with a curved tip to help facilitate passage through the bladder neck in the presence of obstruction from an enlarged prostate gland.^4^ In a 2011 American Urologic Association update, Villaneuva and Hemstreet stated that after two failed attempts using the aforementioned techniques they would consider using a hydrophilic guidewire to facilitate catherization.^3^

In the case presented here we used ultrasound (US) to guide the placement of a hydrophilic guidewire into the bladder and subsequently guide the catheter over the guidewire and into the bladder. We ultimately confirmed the placement of the catheter using US. To the best of our knowledge, there have been no previous studies or case reports in the emergency medicine (EM) literature related to the use of a guidewire for the placement of a transurethral catheter under US guidance.

## CASE REPORT

An 82-year-old male with a history of benign prostatic hypertrophy (BPH) and urinary retention with prior placement of a suprapubic catheter presented with a chief complaint of worsening abdominal pain and distension. The previous suprapubic catheter had been removed by an outside facility five days prior to presentation. On arrival to the emergency department (ED) he was complaining of 7/10 lower abdominal pain and the inability to urinate. The rest of the review of systems was negative and the only pertinent past medical or surgical history was BPH. He denied any substance abuse and denied taking any medications besides anti-hypertensives. On exam, the patient appeared very uncomfortable, had a mildly distended bladder, and significant suprapubic tenderness with no rebound or guarding. A bladder scan was performed at bedside, which revealed 560 milliliters of urine. After a failed attempt to place a 14 French Foley catheter the nurse attempted to catheterize the patient with a coudé tip catheter. This procedure was also unsuccessful and the patient reported increased pain and discomfort.

**Image 1:**
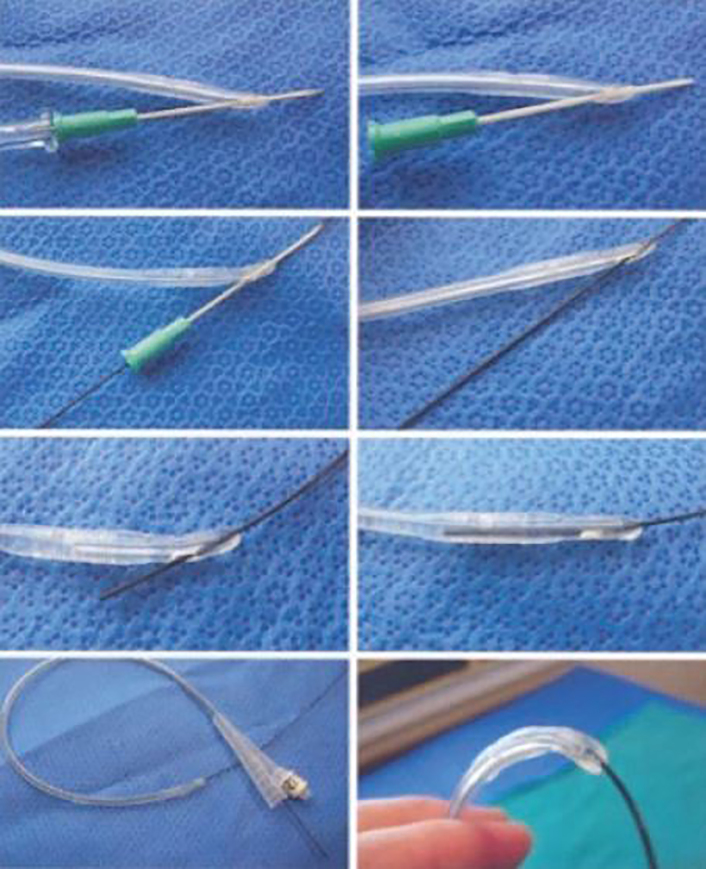
Blitz technique. 7 Using an 18 gauge angiocatheter, puncture a small hole at the tip of the urinary catheter. Then thread the guidewire through the side hole that was already present on the catheter and the hole that was just created at the tip of the urinary catheter. (Used kindly with permission from Dr. Villanueva.)

**Image 2:**
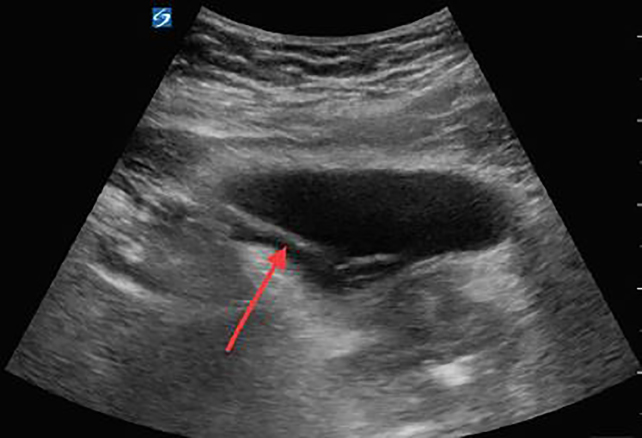
A transabdominal ultrasound image of the urinary bladder in transverse view demonstrating a hyperechoic line (red arrow) representing the guidewire entering the bladder.

**Image 3:**
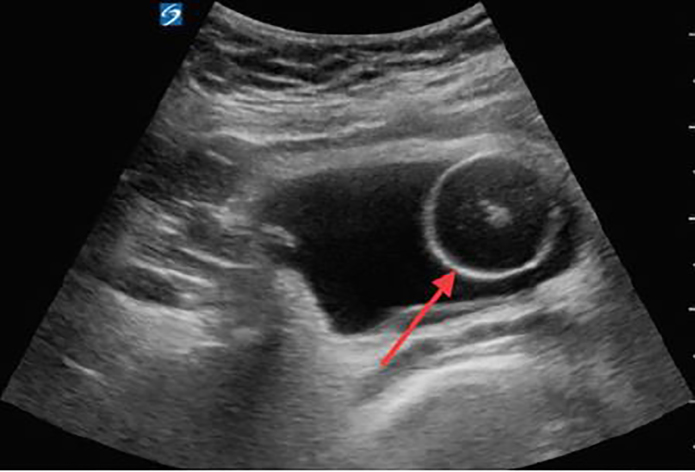
Transabdominal ultrasound, in transverse, demonstrating a Foley catheter with balloon inflated (red arrow) in the right side of the urinary bladder

We chose to place a 16 French Foley catheter over a guidewire using the Blitz technique (Image 1).^5^ A portable US machine, the SonoSite X-Porte with the curvilinear probe (model C60xp with a bandwidth of 5-2MHz) was used during the placement of a guidewire, the ZIPwire^TM^ Hydrophilic Guidewire (Boston Scientific) through the urethra into the bladder with real-time direct visualization. After confirmation of the guidewire in the bladder on US (Image 2), we advanced the catheter over the guidewire, using the Seldinger technique. Using US again, we watched the Foley catheter enter the bladder (Image 3) and the balloon inflate in the appropriate location. The guidewire was removed and 700 milliliters of clear yellow urine was obtained in the collection bag. The patient’s discomfort resolved and he was discharged to home with the catheter in place and a urology appointment for follow-up.

## DISCUSSION

As emergency physicians (EP) we commonly encounter urinary retention. In two large cohort studies of men in the United States age 40 to 83 years old, the overall incidence was 4.5 to 6.8 per 1,000 men per year. The incidence dramatically increases with age so that a man in his seventies has a 10% chance and a man in his eighties has a more than 30% chance of having an episode of acute urinary retention.^6^,^7^ The treatment of urinary retention is transurethral catheterization. Current EM teaching does not offer much guidance for managing difficult catheterizations, and urology consultation is recommended when a transurethral catheter does not provide adequate bladder drainage. Knowledge of this topic remains sparse in both EM and nursing specialties, and recommendations are seldom supported by evidence-based research.^8^

**CPC-EM CAPSULE**

What do we already know about this clinical entity?

Urologists have been using hydrophilic guidewires in cases of difficult catheter placement for many years. These tools are not commonly used by emergency physicians (EP).

What makes this presentation of disease reportable?

Difficult urinary catheterization is a common occurrence in the emergency department. Hydrophilic guidewire use with ultrasound confirmation has not been reported.

What is the major learning point?

By using a guidewire and point-of-care ultrasound, the EP can safely place a Foley catheter in cases of difficult urinary catheterization.

How might this improve emergency medicine practice?

Urinary retention is common and it is important for EPs to have various tools at their disposal to achieve successful catheterization.

Urinary catheterization may be associated with complications such as traumatic insertion, creation of false tracts, urethral trauma, clogging of the catheter, and accidental or intentional removal of a urinary catheter with the balloon inflated.^3^ The most common injury sites are the posterior and bulbous urethra, and the most frequently reported injuries are false passages created by forceful catheterization, as well as mucosal and submucosal tissue tears caused by balloon inflation in an improper position in the urethra.^3^,^9^ These complications may ensue even in consultation with experienced urologists. No matter what the cause, urinary catheterization is an inherently uncomfortable procedure. Multiple attempts can result in significant patient distress, as well as bleeding, urethral trauma and increased healthcare costs to both the patient and the hospital.

Chavez et al. performed a retrospective chart review from 1998 to 2007, which looked at the costs of a traumatic urethral catheterization. The authors found that of 221,045 patients who underwent urethral catheterization, 3,101 (1.4%) were traumatic. The incidences of urinary tract infection, cystitis and septicemia two weeks after catheter induced urethral injury were 12.72%, 3.45% and 1.9% with estimated costs of $11,052, $482 and $48,935, respectively.^10^

The description of guidewire use during catheterization was first published in 1989 by SJ Krikler, yet urologists had been using this technique for years.^11^ The first attempts were performed using cystoscopy to insert the guidewire.^12^ Since then, urologists have been using hydrophilic guidewires without the assistance of a cystoscope to place catheters in situations where other attempts were unsuccessful or resulted in trauma.^3^, ^13^–^16^ The technique is performed by blindly inserting the guidewire approximately half of the total length of the guidewire (150 centimeters). The Foley catheter is prepared using the Blitz technique (Image 1). Using this technique, the clinician punctures a hole at the tip of the Foley catheter using an 18-gauge needle. He then threads the Foley catheter over the guidewire using the hole created at the tip. The Foley catheter can then be advanced over the guidewire until it reaches the bladder. The guidewire can then be removed; urine returned from the catheter confirms proper position.^13^

Freid et al. examined the safety and efficacy of this blind introduction of a guidewire into the bladder and reported success in 19 of 20 attempts and no complications associated with the procedure.^16^ One of the major disadvantages and potential complications of attempting to introduce a guidewire into the bladder is the potential for misplacement.^3^ This can potentially be alleviated by confirming placement of the guidewire into the bladder using US guidance. A hydrophilic guidewire ranges in diameter from 0.018 to 0.038 inches in diameter with a soft, flexible tip that ranges from 3–15 centimeters in length. The total length of the guidewire is usually 150 centimeters. The shaft of the wire is rigid and constructed in two layers, an inner core and an outer coating made up of a hydrophilic polymer, most commonly polytetrafluoroethylene. The hydrophilic coating provides the low-friction characteristics necessary to navigate strictures or a hypertrophied prostate gland.

A number of EPs use point-of-care ultrasound to guide or confirm correct placement of a urinary catheter,^17^ but performing the Blitz technique under US guidance has not been described in the EM literature. This is a useful technique for any EP to have in his repertoire and will most certainly decrease delays in catheter placement, traumatic catheter insertions, urethral trauma, and late-night consults to the urologist for patients presenting to the ED with urinary retention.

## CONCLUSION

Physicians should be aware of the risks and common pitfalls associated with transurethral catheterization. They should also be familiar with the procedure described above in which we were able to successfully place a Foley catheter using ultrasound for guidewire placement in a patient with multiple previous failed attempts. By using this technique we hope that EPs can successfully place a difficult Foley catheter, thus avoiding a consult to the urologist for placement.

Documented patient informed consent and/or Institutional Review Board approval has been obtained and filed for publication of this case report.
